# Metatranscriptomic Signatures Associated With Phytoplankton Regime Shift From Diatom Dominance to a Dinoflagellate Bloom

**DOI:** 10.3389/fmicb.2019.00590

**Published:** 2019-03-22

**Authors:** Yaqun Zhang, Xin Lin, Xinguo Shi, Lingxiao Lin, Hao Luo, Ling Li, Senjie Lin

**Affiliations:** ^1^State Key Laboratory of Marine Environmental Science, College of Ocean and Earth Sciences, Xiamen University, Xiamen, China; ^2^College of Biological Science and Engineering, Fuzhou University, Fuzhou, China; ^3^Department of Marine Sciences, University of Connecticut, Groton, CT, United States

**Keywords:** harmful algal blooms, metatranscriptome, *Prorocentrum donghaiense*, rhodopsin, energy generation, diatoms

## Abstract

Diatoms and dinoflagellates dominate coastal marine phytoplankton communities as major players of marine biogeochemical cycles and their seasonal succession often leads to harmful algal blooms (HABs). What regulates their respective dominances and the development of the HABs remains elusive. Here we conducted time-sequential metatranscriptomic profiling on a natural assemblage that evolved from diatom dominance to a dinoflagellate bloom to interrogate the underlying major metabolic and ecological drivers. Data reveals similarity between diatoms and dinoflagellates in exhibiting high capacities of energy production, nutrient acquisition, and stress protection in their respective dominance stages. The diatom-to-dinoflagellate succession coincided with an increase in turbidity and sharp declines in silicate and phosphate availability, concomitant with the transcriptomic shift from expression of silicate uptake and urea utilization genes in diatoms to that of genes for light harvesting, diversified phosphorus acquisition and autophagy-based internal nutrient recycling in dinoflagellates. Furthermore, the diatom-dominant community featured strong potential to carbohydrate metabolism and a strikingly high expression of trypsin potentially promoting frustule building. In contrast, the dinoflagellate bloom featured elevated expression of xanthorhodopsin, and antimicrobial defensin genes, indicating potential importance of energy harnessing and microbial defense in bloom development. This study sheds light on mechanisms potentially governing diatom- and dinoflagellate-dominance and regulating bloom development in the natural environment and raises new questions to be addressed in future studies.

## Introduction

Diatoms and dinoflagellates are two ecologically important groups of eukaryotic phytoplankton in the ocean, representing major contributors of marine primary production, which participates in the global carbon fixation and O_2_ production. They are major players in biogeochemical cycles of elements (including biological pump of carbon for deep-sea sequestration) and coastal seasonal succession (Margalef, 1978; Falkowski et al., 2004). While diatoms normally dominate, dinoflagellates can outgrow them seasonally or on occasions of environmental perturbations, often resulting in harmful algal blooms (HABs) that exact devastating impacts on the marine ecosystem, economy, and public health. How these two keystone phytoplankton groups are so successful and how their “seesaw” dynamics tips toward a bloom are fundamental yet poorly understood marine ecology questions.

Numerous physioecological studies have shown that diatoms and dinoflagellates have distinct ecological niches (Irwin et al., 2012). Diatoms are generally known for a high capacity to assimilate silicate and nitrogen and prefer lower temperatures, as such tend to thrive in cool nitrate-rich environments (Lomas and Glibert, 2000; Falkowski et al., 2004; Malviya et al., 2016). In contrast, dinoflagellates tend to bloom in warm waters, which are usually more nutrient poor (Smayda, 1997). Some studies also demonstrate that dinoflagellate red tide blooms increased as the N:P ratio fell (Anderson et al., 2002), while some other studies show that species differentiate in nutrient uptake efficiency and favorable nutrient conditions (Glibert and Burkholder, 2011). The ample ecological observations and experimental data constitute the core of our understanding on the relationship between diatoms and dinoflagellates (Anderson et al., 2002; Falkowski and Oliver, 2007). Yet it is still difficult to pinpoint which metabolic processes underpin the differential niches, govern their respective dominances and regulate the diatom-to-dinoflagellate succession leading to the dinoflagellate bloom, largely because *in situ* metabolic profiles are generally difficult to measure and contributions of different taxa are difficult to partition (Alexander et al., 2015a).

The metatranscriptomic approach has proven to be powerful in characterizing metabolic configurations in dinoflagellates (Lin et al., 2010; Cooper et al., 2014; Zhuang et al., 2015; Gong et al., 2017), diatoms (Alexander et al., 2015a), and other lineages (Alexander et al., 2015b) in the natural marine environment. In this approach, the relative expression levels of genes in the community are indicative of relative activities of the biochemical or physiological phenotypes regulated by the proteins or enzymes encoded by those genes. Furthermore, these genes can be traced to the organisms existing in the community by sequence comparison so that the contributing taxa to the active phenotypes can be identified and the relative contribution of each taxon can be assessed. Here we employed time-sequential (month long) metatranscriptomics to characterize the evolution of a phytoplankton community from diatom dominance (dominantly *Skeletonema*) to dinoflagellate (*Prorocentrum donghaiense*) bloom, an annual event observed in the studied area in East China Sea (ECS) for more than a decade (Liu et al., 2016; Xiao et al., 2017). The metatranscriptomic investigation was conducted to examine three hypotheses regarding the diatom-to-dinoflagellate regime shift: (1) Diatoms and dinoflagellates may use the same biological processes (e.g., energy production, nutrient acquisition, and stress protection) while maintaining their respective dominance in the natural assemblages; (2) diatoms and dinoflagellates may use distinct metabolic pathways in their respective dominance in response to environmental variations; and (3) Competitive advantage-conferring biochemical pathways may be facilitative for dinoflagellates to outgrow diatoms and form blooms.

## Materials and Methods

### Field Campaign, Environmental Measurements, and Sample Collection

Because regime shift from diatom dominance to a dinoflagellate bloom (most frequently of *P. donghaiense*) has occurred in the April–May period in the ECS coastal water since 1990s (Gao and Shao, 2011; Sun et al., 2017; Supplementary Figure S1), a research cruise was launched in the high HAB risk area of the Yangtze River Estuary, ECS. Surface seawater (0–2 m) samples were collected from April 30 to May 20 in 2014 when the phytoplankton community underwent major changes. Station 7 (29°1′0″N, 122°9′27″E) was sampled on April 30 (T0) and May 20 (T3) whereas station 7A (29°3′19″N. 122°16′30″E) on May 13 (T1) and May 15 (T2) (Supplementary Figure S1). Fifty-mL seawater samples were collected for each time point and fixed in Lugol’s solution (2%) for subsequent microscopic species identification and enumeration in Sedgwick-Rafter counting chamber. For metatranscriptomic analyses, 4–13L seawater samples were prefiltered through a 200 μm nylon mesh to remove large particles and zooplankton, then filtered through 3 μm pore-size 144 mm diameter polycarbonate membrane (Merck Millipore, MA, United States) using a vacuum pump under low vacuum pressure (<10 PSI). The filters were cut into four even pieces, and each was immediately transferred to a 2 mL tube; two tubes containing 1 mL TRI Regent buffer for RNA extraction and two containing 1 mL DNA lysis buffer (100 mM Tris-Cl, 50 mM EDTA, pH = 8) for DNA analysis to be reported elsewhere. To minimize changes of gene expression and community structure in the process, total sample processing time, from arrival on deck to the buffers, was no more than 15 min. The samples for RNA were frozen in liquid N_2_ where they were kept until the end of the cruise, and once transported back to our laboratory the samples were stored at -80°C until RNA extraction. The samples for DNA were stored at -20°C until DNA extraction. Temperature, salinity, and turbidity were measured using CTD (SBE 17plus V2, Sea-Bird Scientific, United States) at each sampling event. Concentrations of dissolved inorganic nitrogen (DIN) (ammonium, nitrate, and nitrite), silicate and phosphate were determined using continuous flow analyzer, all measurements were performed on triplicate samples San^++^ (SKALAR, Breda, Holland) (Li et al., 2017).

### RNA Extraction and Illumina High-Throughput Sequencing

Frozen samples were thawed and plankton cells were washed off the filters using pipettes in RNase-free petri dish and then moved back into the 2 mL centrifuge tube and centrifuged at 14000 × *g* for 15 s, and most of the supernatant was removed into a fresh tube leaving only about 200 μL behind. Next, a 1:1 mixture of 0.5 mm and 0.1 mm-diameter glass beads (Biospec, Bartlesville, OK, United States) were added to the 200 μL sample, with the volume of beads being approximately equal to the cell pellets. Then the samples with beads were loaded onto a FastPrep-24 bead mill (MP Biomedicals, Solon, OH, United States) for bead-beating at the rate of 6 m/s, performed three times with 1 min intervals when the samples were placed on ice. Two microliters of homogenate were checked microscopically to verify complete cell breakage. Then, the removed supernatant was combined with the homogenized component, and RNA was extracted following the TRI Reagent protocol coupled with the Direct-zol^TM^ RNA columns as reported previously (Lin et al., 2010). RNA concentration was measured using a NanoDrop ND-2000 Spectrophotometer (Thermo Fisher Scientific, Walthman, MA, United States), while integrity was assessed using RNA 6000 Nano LabChip Kit in microcapillary electrophoresis Agilent 2100 Bioanalyzer (Agilent Technologies, Santa Clara, CA, United States). The RNA integrity number (RIN) of the samples was all above the recommended value (6.0) for metatranscriptome sequencing. For Illumina RNA-seq sequencing, 1 μg total RNA from each sample was used to isolate mRNA using NEBNext Poly(A) mRNA Magnetic Isolation Module (New England Biolabs, Ipswich, MA, United States). mRNA was then fragmented with First Strand Synthesis Reaction Buffer and Random Primer Mix (2×) at 94°C for 10 min, and first strand cDNA was synthesized using ProtoScript II Reverse Transcriptase and the second-strand cDNA was synthesized using Second Strand Synthesis Enzyme Mix. The double-stranded cDNA purified, end repaired, and ligated to adaptors. Fragments of about 400 bp (with the approximate insert size of 250 bp) were selected and sequenced on Illumina HiSeq instrument (Illumina, San Diego, CA, United States).

### Transcriptome Analysis and Gene Expression Quantification

After Illumina RNA-seq sequencing, low quality reads (cut-off average score value of 20) and adaptors were removed using Trimmomatic V0.30 (Bolger et al., 2014), and then two different ways were taken to process the clean reads. First, to determine the taxonomic origin of the transcripts, clean reads from the time-serial samples were mapped to a “local database” consisting of all the assembled transcriptomes of eukaryotic algae generated by the Marine Microbial Eukaryotic Transcriptome Sequencing Project (MMETSP, downloaded in March, 2015) and *P. donghaiense* transcriptome (Shi et al., 2017) using Bowtie2 version 2.2.1 (parameters: -sensitive) (Langmead and Salzberg, 2012; Alexander et al., 2015a). Reads mapped to diatoms and to *P. donghaiense* were separated for further analysis. Expression levels of these mapped reads were then quantified using HTSeq python 3.4.3 package (Anders et al., 2014), and normalized to total transcriptomic reads of the taxonomic group using FPKM value (Fragment Per Kilo bases per Million reads). Differential gene expression for diatoms and dinoflagellates, separately, was both analyzed between bloom group (T1, T2, T3, which were treated as triplicates for the bloom condition) and non-bloom group (T0). Count-based differential expression for metatranscriptome was analyzed using edgeR Bioconductor package, with TMM normalization (Robinson et al., 2010), and significance of differential expression was assigned with edgeR’s exactTest function following previous reports (Marchetti et al., 2012; Shi et al., 2017). To strengthen reliability of differential gene expression profile found from the dataset, which lacked replicates at T0, NOISeq-sim (Tarazona et al., 2015) (Tarazona et al., 2011) was also conducted to independently identify the differential expressed genes (*q* = 0.9). Only the genes identified by both edgeR and NOISeq-sim as differentially expressed were accepted as differential expressed genes. The criteria for defining statistically significant differential expression were fold changes >2 and FDR <0.05 (adjusted *P*-value, determined by the Benjamini and Hochberg multiple-testing correction implemented in the “p. adjust” method in R).

Second, to retrieve all expressed genes, including those not represented in the MMETSP database, our RNA-Seq reads were assembled *de novo* using Trinity (r2013-02-25) (parameter: -default), and the assembled sequences were clustered to remove redundancy using software TGICL (TIGR Gene Indices clustering tools, V2.1) (Pertea et al., 2003). Open reading frames were predicted and confirmed based on Markov model. The resulting unigenes were used for BLAST search and annotation against the NCBI non-redundant (nr) database^1^ and Swiss-Prot with a 1e-5 value cutoff, and also annotated against eggNOGV4.1 database (for COG annotation) with a 1e-5 value cutoff and PFAM database with a 1e-3 value cutoff. KEGG mapping and analysis were carried out on KEGG Automatic Annotation Server (KAAS).

### Phylogenetic Analyses

Phylogenetic analysis was conducted to assess taxonomic affiliation of particular genes. Deduced protein sequences and selected reference sequences were aligned using ClustalW in MEGA 6 (Larkin et al., 2007; Tamura et al., 2013). ProtTest 3.4.2 were used to find the best model of protein evolution (Darriba et al., 2011). Phylogenetic trees were inferred using Maximum likelihood method in MEGA 6 using the model with rates and parameters estimated from ProtTest, with 1000 bootstrap replicates performed to obtain statistical support for the tree topology. The resulting tree file from MEGA 6 was then uploaded to the iTOL to make further modifications (Letunic and Bork, 2007).

### Reverse Transcription Quantitative PCR (RT-qPCR) of Rhodopsin

As rhodopsin was found to be one of the highly expressed genes during the bloom, *P. donghaiense* rhodopsin expression was quantified for the field samples and additional samples collected from cultures for comparison. *P. donghaiense* cultures (triplicate) were grown at 20°C under a photon flux of 100 μE m^-2^ s^-1^ with a 14:10 h light/dark cycle in L1 medium (without silicate) with 36 μM phosphate concentration. When the cultures entered the exponential growth stage, samples were collected at the middle of light period. RNA extraction was carried out as described above. For both the field and the culture samples, 300 ng of total RNA of each sample were reverse-transcribed in a final volume of 20 μL using a PrimeScript RT reagent kit with gDNA Eraser (Perfect Real Time) (Takara Biotechnology, Dalian, China) including a gDNA removal step. Specific primers of rhodopsin were designed based on the unigene sequences obtained from *P. donghaiense* transcriptome (Supplementary Table S1). We used calmodulin and actin as reference genes to normalize the expression of the selected genes as reported (Shi et al., 2013) (Supplementary Table S1). RT-qPCR was performed on iCycle iQ Real-Time PCR Detection System using Bio-Rad iQ SYBR Green Supermix Kit (Bio-Rad Laboratories, Hercules, United States). Relative expression was calculated using the comparative Ct method (2^-ΔΔCt^) (Livak and Schmittgen, 2001). Significant differences of environmental factors (e.g., silicate, PO_4_^3-^) between the means of T0 and T1, T2, T3 and RT-qPCR between the means of the four samples were determined using independent samples *t*-test (two sided).

### Data Availability

Raw metatranscriptome sequencing reads in our study were deposited in NCBI Sequence Read Archive under accession numbers SAMN06849052, SAMN06849053, SAMN06849054, and SAMN06849072.

## Results and Discussion

### Community Regime Shift From Diatom Dominance to Dinoflagellate Bloom and Changes of Environmental Conditions

**FIGURE 1:**
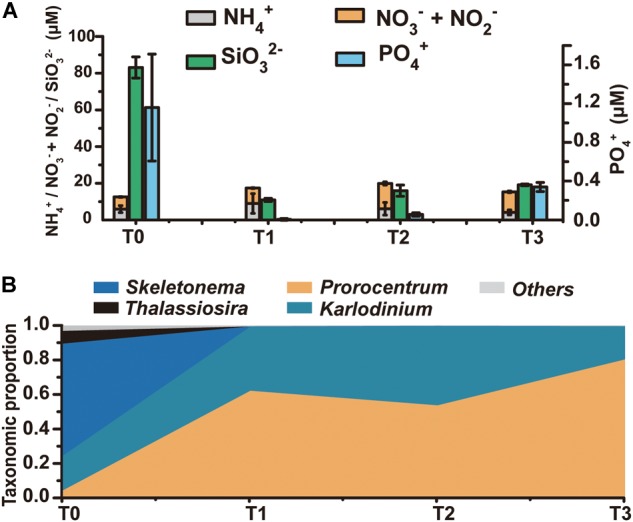
The dynamics of the phytoplankton community and the environmental conditions from T0 to T3. **(A)** Concentrations of major nutrients in the ambient environment, including various forms of nitrogen, silicate, and phosphate. **(B)** Taxonomic distribution (fractions) of transcripts in the metatranscriptome (for the mapped reads) at each time point.

A research cruise was conducted from April 23 till May 23, 2014 to follow the regime shift from diatom dominance to a dinoflagellate bloom in the ECS coastal area, covering a series of stations along three transects (Supplementary Figure S1). At T0 (April 30) no bloom was noticed and microscopic examination indicated a diverse phytoplankton community dominated by the diatoms *Skeletonema* and *Pseudo-nitzschia*. A bloom was visible from T1 through T3 (May 13–20) and microscopic check indicated that the bloom was caused by the dinoflagellate *P. donghaiense* (Supplementary Figure S1). We chose four time points to represent a regime shift from dominance by diatoms (T0) to a bloom predominated by the dinoflagellate *P. donghaiense* (T1, T2, and T3, on May 13, 15, and 20, respectively). Total eukaryotic phytoplankton concentration at T0 was 5.23 × 10^5^ cells L^-1^, 88.64% of which was contributed by diatoms (64.98% by *Skeletonema* and 20.62% by *P. nitzschia*), whereas that at T1, T2, and T3 was 2.54 × 10^6^, 4.89 × 10^6^, and 10.34 × 10^6^ cells L^-1^, respectively, with 79.74, 77.45, and 90.96% accounted for by dinoflagellates, mostly *P. donghaiense* (69.32%, 67.26%, 86.04%, respectively) (Supplementary Figure S2). As a major nutrient for diatom growth, silicate concentration decreased markedly, from 83.11 μM at T0 to <20 μM at T1, T2, and T3 (*t*-test, *P* < 0.05, *n* = 3) (Figure 1A). DIN remained at a high concentration (>10 μM) throughout the study period (Figure 1A). In contrast, PO_4_^3-^ concentration decreased dramatically from 1.16 μM at T0 to 0.34 μM or lower subsequently (*t*-test, *P* < 0.05, *n* = 3) (Figure 1A). The turbidity of the water increased remarkably (from 0.51FTU to 11.02FTU, surface seawater temperature increased from 16.50 to 19.19°C from April 30 to May 13–20, and salinity decreased slightly from 30.63 to 28.51 PSU in this period, Supplementary Figure S3).

### Common Metabolic Pathways Underlying Diatom- and Dinoflagellate-Dominance

HiSeq RNA sequencing generated 69–96 million clean reads from T0, T1, T2, and T3 samples, which were, respectively, assembled into 416,844, 263,750, 249,364, and 179,699 unigenes (Supplementary Table S2). To assess the expression levels of genes in each major lineage of phytoplankton, we mapped the metatranscriptome clean reads to eukaryotic algal and *P. donghaiense* transcriptome databases (Keeling et al., 2014; Shi et al., 2017), and calculated the proportion of reads mapped to that lineage out of total reads that were mapped to the database. The number of reads mapped to diatoms was 3.87 × 10^6^, 1.06 × 10^5^, 1.47 × 10^5^, 1.92 × 10^4^ for T0, T1, T2, and T3 samples, and the number of reads mapped to dinoflagellates was 1.24 × 10^6^, 2.75 × 10^7^, 2.60 × 10^7^, 4.34 × 10^7^ at these four time points. Furthermore, the T0 library mainly binned with *Skeletonema*, which accounted for 65% of the mapped reads, while T1, T2, and T3 libraries were dominated by dinoflagellates, with the proportion of *Prorocentrum* transcripts growing from 4% at T0 to 54–80.32% later (Figure 1B). The large proportion of diatom transcripts at T0 and that of dinoflagellate transcripts at the T1-T2-T3 period were consistent with the trend of diatom and dinoflagellate abundances shown above, indicating coincidence between transcriptional activity and numerical abundance of the lineages.

**FIGURE 2:**
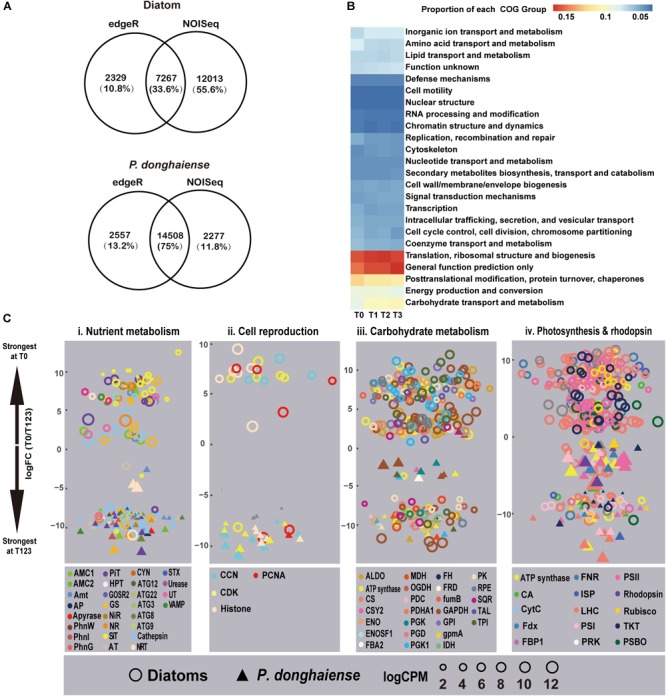
Metatranscriptomic outlook in diatoms and *P. donghaiense*. **(A)** Number of differential expressed genes determined by edgeR and NOISeq. **(B)** Clusters of Orthologous Groups comparison over the four time points. **(C)** T0 vs. T123 fold changes in transcript abundances in four major metabolic pathways (FDR <=0.05). Data on top were most strongly expressed at T0 whereas those at bottom most strongly expressed at T123. Solid triangles, *P. donghaiense*; hollow circles, diatoms. Color depicts a gene or gene group. Symbol size depicts the expression level (log CPM [counts per million]). Gene abbreviations: **(i)** AMC1/2, metacaspase-1/2; Amt, ammonium transporter; AP, alkaline phosphatase; AT, amino acid transporter; ATG, autophagy-related protein; CYN, cyanate hydratase; GOSR2, glogi SNAP receptor complex member 2; GS, glutamine synthetase; HPT, high-affinity phosphate transporter; NiR, nitrite reductase; NR, nitrate reductase; PhnG, 6-phosphofructokinase 7; NRT, nitrate transporter 2.5; PhnI, uncharacterised protein (PhnI domain); PhnW, 2-aminoethylphosphonate-pyruvate transaminase; PiT, phosphate transport protein; SiT, silicon transporter; STX, syntaxin; UT, urea transporter; VAMP, synaptobrevin homolog. **(ii)** CCN, Cyclin; CDK, cyclin-dependent kinase; PCNA, proliferating cell nuclear antigen. **(iii)** ALDO, Fructose-bisphosphate aldolase class 1; CS, citrate synthase; CSY2, citrate synthase 2 mitochondrial; ENO: enolase; ENOSF, mitochondrial enolase superfamily; FBA2, fructose-bisphosphate aldolase 2, chloroplastic; FH, fumarate hydratase, mitochondrial; FRD, fumarate reductase; FumB, fumarate hydratase class **(i)** anaerobic; GAPDH, glyceraldehyde-3-phosphate dehydrogenase; GPI, glucose-6-phosphate isomerase; GpmA, 2, 3-bisphosphoglycerate-dependent phosphoglycerate mutase; IDH, isocitrate dehydrogenase; MDH, malate dehydrogenase; OGDH, 2-oxoglutarate dehydrogenase mitochondrial; PDC: pyruvate dehydrogenase [NADP(+)], mitochondrial; PDHA1, pyruvate dehydrogenase, mitochondrial; PGD, 6-phosphogluconate dehydrogenase decarboxylating 2; PGK, phosphoglycerate kinase; PGK1, phosphoglycerate kinase chloroplastic; PK, pyruvate kinase; RPE, ribulose-phosphate 3-epimerase; SQR, succinate dehydrogenase flavoprotein subunit 1 mitochondrial; TAL, transaldolase; TPI, triosephosphate isomerase. **(iv)** CA, Carbonic anhydrase; CytC, cytochrome c6; FBP1, fructose-1 6-bisphosphatase; Fdx, ferredoxin; FNR, ferredoxin-NADP^+^ reductase; ISP, cytochrome b6-f complex iron-sulfur subunit, chloroplastic; LHC, light harvesting protein complex; PRK, phosphoribulokinase; PSBO, oxygen-evolving enhancer protein 1; PSI/PSII, photosystem I/II proteins; Rubisco, ribulose-1, 5-bisphosphate carboxylase/oxygenase; TKT, transketolase.

Using both edgeR and NOISeq analysis in combination, an analysis strategy to enhance the reliability of identified differentially expressed genes (DEGs), 7,267 (15.87%) genes out of 45,788 mapped genes in diatoms and 14,508 (12.77%) genes out of 11,3599 mapped genes (Bowtie2, parameters: -sensitive) in *P. donghaiense* were identified as DEGs (Figure 2A) between diatom-dominant assemblage and dinoflagellate-bloom assemblages. However, from the functional perspective, the whole phytoplankton community showed relatively stable COG functional group landscape over time (Figure 2B). Furthermore, there was a high similarity among T1, T2, and T3 in ambient environmental conditions, phytoplankton community structure, and the metatranscriptomic profile. Principal component analysis (PCA) of FPKM across the four time points also showed that gene expression profile at T0 was markedly different from that at T1, T2, and T3, while profiles from T1, T2, and T3 were similar (Supplementary Figure S4). Therefore, differential gene expression was further analyzed by treating data from these three time points as replicates and this time period is referred to as T123 hereafter. Interestingly, diatoms at T0 and dinoflagellates at T123 showed a similar set of up-regulated gene repertoires, e.g., ones that are involved in energy generation, carbohydrate metabolism, nutrient uptake and assimilation, and cell proliferation (Figure 2C). This result provides circumstantial support to our first hypothesis that diatoms and dinoflagellates may use the same biological processes to maintain their dominance. However, as will be elaborated later, diatoms and dinoflagellates appeared to use distinct metabolic pathways in respective dominant or bloom condition (Figure 2C and Supplementary Dataset 1), in support of our second hypothesis.

Both the diatom-dominant and dinoflagellate-bloom assemblages exhibited up-regulation, relative to their respective non-dominant periods, of genes regulating uptake and assimilation of N nutrient, such as ammonium transporter, nitrate transporter (NRT), nitrate/nitrite reductase, and glutamine synthetase (GS) (Figure 2Ci). This suggests that there might be DIN input allowing utilization by the phytoplankton community while keeping the ambient concentration relatively stable in the study period. A notable exception was homologs of NRT and GS from *P. nitzschia* sp., which showed up-regulation at T123. This, along with the up-regulation of a cyclin-dependent kinase, is indicative of active nutrient uptake and growth of this diatom at T123. This attests to the recently documented differential niches among diatom species in the natural assemblages (Alexander et al., 2015a).

An algal bloom can result from cell proliferation or grazing depression, the relative importance of which is often debated (Irigoien et al., 2005). For both the diatom dominance and the *P. donghaiense* bloom, cell proliferation was speculated to be active because genes encoding cell cycle proteins such as PCNA were all up-regulated in diatoms at T1 and in *P. donghaiense* at T123 (Figure 2Cii and Supplementary Dataset 1). Besides, G2/mitotic-specific cyclin-B was also up-regulated in *P. donghaiense* at T123. PCNA and G2/mitotic-specific cyclin-B have been shown to be associated with mitosis and cell proliferation in some dinoflagellates (Toulza et al., 2010; Zhuang et al., 2013).

We found that the most strongly regulated synthase and redox genes at T0 were those encoding damage repair and antioxidants in diatoms, e.g., geranylgeranyl diphosphate reductase (CHLP), peptide methionine sulfoxide reductase (MsrA), superoxide dismutase (SOD), and thioredoxin (Supplementary Figure S5a and Supplementary Dataset 2). Many of these supply reducing power for detoxifying lipid hydroperoxides or regulate repair of oxidized proteins (Ramel et al., 2009). *P. donghaiense* bloom metatranscriptome also contained anti-stress genes (antioxidants and UV filters) at T123 (Supplementary Figure S5b and Supplementary Dataset 2). Some of these, e.g., CHLP, SOD, and thioredoxin, were shared with the T0-diatom community (Supplementary Dataset 2 and Supplementary Figure S5), while others, e.g., 2-epi-5-epi-valiolone synthase for the biosynthesis of the UV-filtering mycosporine-like amino acid (MAA) shinorine (Lemmers et al., 2010) and flavonol synthase for the biosynthesis of UV combating flavonoids (Samanta et al., 2011) were unique in the *P. donghaiense* bloom (Supplementary Dataset 2). The elevated expression of the anti-stress genes during both diatom and dinoflagellate dominance is consistent with the importance of anti-oxidative stress in N metabolism and many cellular activities (Rosenwasser et al., 2014).

### Unique Transcriptomic Signatures in Diatom Dominance

We further looked for specific metabolic pathways in diatoms that may be associated with their dominance to gain more support for our second hypothesis. As described below, a set of pathways were found to be strongly associated with diatom dominance.

#### Up-Regulated Si and Urea Uptake Mechanisms

Two types of silicate transporter genes in diatoms were identified, which shared high sequence similarity with *Cylindrotheca fusiformis* SiTs (SiT gene clade A) and *Skeletonema costatum* SiTs (SiT gene clade E) (Durkin et al., 2016). Both of them were more highly expressed at T0 than T123 (Figure 2Ci). It was previously shown that most types of SiTs in *C. fusiformis* (SiT gene clade A) were up-regulated to a maximum level around 2 h and 20 min after silicon addition (Hildebrand et al., 1998). Thus the high expression of SiTs indicated the active silicate uptake at T0. Besides, diatoms exhibited elevated capacity of urea uptake at T0, as indicated by the markedly up-regulated genes of urea active transporter 1 and urease (Figure 2Ci).

#### High Expression of Carbohydrate Metabolic Pathway

**FIGURE 3:**
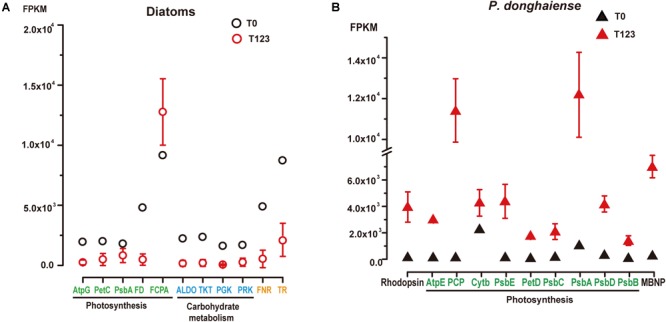
Expression levels of representative highly expressed genes in **(A)** diatoms and **(B)** *P. donghaiense* between T0 and T123. AtpG, Atp synthase gamma chain; PetC, cytochrome b6-f complex iron-sulfur subunit; PsbA, photosystem II D1; FD, ferredoxin; FCPA, fucoxanthin-chlorophyll *a*-*c* binding protein; ALDO, fructose-bisphosphate aldolase; TKT, transketolase; PGK, phosphoglycerate kinase; PRK, phosphoribulokinase; FNR, ferredoxin-nadp reductase embryo isozyme; TR, trypsin; TR-II, trypsin 5g1; AtpE, chloroplast ATP synthase subunit C; PCP, chloroplast peridinin-chlorophyll *a*-binding protein precursor; PsbE, cytochrome b559 subunit alpha; PetD, cytochrome b6-f complex subunit IV; PsbC, photosystem II CP43 reaction center protein; PsbD, photosystem II protein D2; PsbB, photosystem II CP47 reaction center protein; MBNP, major basic nuclear protein.

For diatoms and *P. donghaiense*, genes involved in carbohydrate metabolism were both upregulated during their dominance (diatoms at T0 compared to T123, *P. donghaiense* at T123 compared to T0) (Figure 2Ciii). However, the expression level of genes involved in carbohydrate metabolism in diatoms at T0 was much higher (FPKM >10^3^, Figure 3A) than the expression level of their homologs in *P. donghaiense* at T123 (FPKM = 83.93–128.66). A previous study also showed overwhelming dominance of carbohydrate metabolism in natural diatom populations (Alexander et al., 2015a).

#### Unsuspected Highly Active Expression of Trypsin

The most conspicuous and surprising signature of the diatom dominance is the very high expression of trypsin (Figure 3A). Two major groups of trypsin (trypsin I and trypsin II) cDNAs were retrieved from the metatranscriptomes, and their representative members were found to phylogenetically affiliate with homologs in *Skeletonema* and *Thalassiosira* (Supplementary Figure S6). They collectively accounted for 1% of the total diatom transcript reads at T0 (FPKM = 8441) while only 0.1–0.6% at T123 (Supplementary Dataset 3). Notably, we found 19 trypsin genes in the genome of the diatom *Thalassiosira pseudonana* (Armbrust et al., 2004) but only two in the dinoflagellate *Fugacium kawagutii* (Lin S. et al., 2015) despite the much smaller genome in the former. The gene copy number and expression level in concert suggest that trypsin may play important roles in diatom growth. Abundant protease genes have been associated with silica nanostructure development in diatoms (Otzen, 2012), which is essential for diatom proliferation. Mainly cleaving peptide bonds with arginine or lysine, trypsin can produce amine-rich residues to promote the production of polyamines, which are required in silica morphogenesis in diatoms (Sumper and Kröger, 2004). This possibility, albeit consistent with the up-regulated expression of Si transporter at T0, needs to be investigated in the future.

### Unique Transcriptomic Signatures in *P. donghaiense* Bloom

With the changes of environmental conditions, increased transcriptional activities of photoenergy acquirement and alternative nutrient assimilation pathways in the dinoflagellate subcommunity distinguished the T123 assemblage from the T0 assemblage (Figure 3). This provides additional support to our second hypothesis about distinct metabolic pathways that might be associated with the dominant states of diatoms and dinoflagellates. Furthermore, this result revealed up-regulated genes of *P. donghaiense* during the dinoflagellate bloom, with predicted functions likely conferring competitive advantages for this dinoflagellate to outgrow diatoms and form a bloom, which is in support of our third hypothesis.

#### Proton-Pump Rhodopsin

**FIGURE 4:**
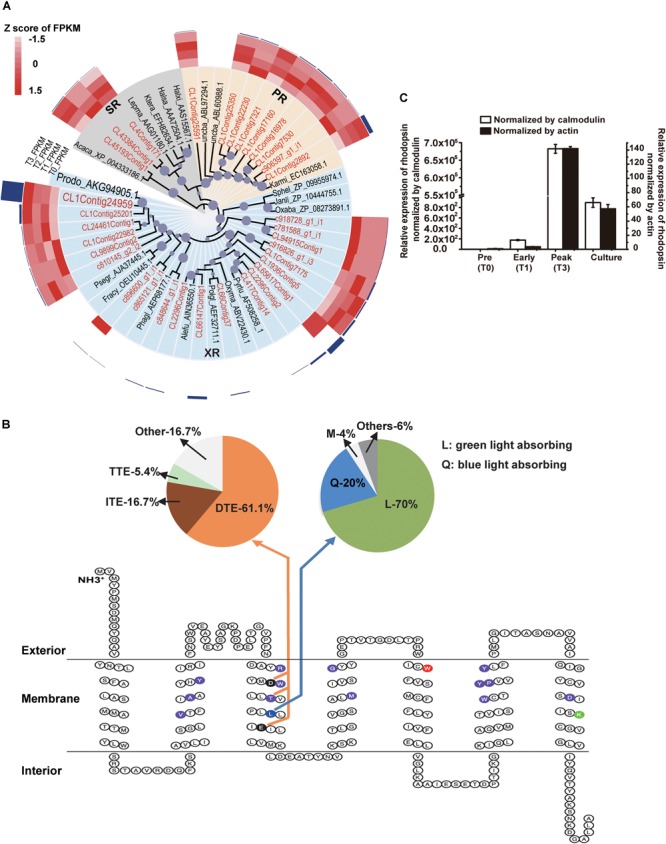
Diversity and expression profile of rhodopsin genes represented in the metatranscriptomes. **(A)** Phylogenetic analysis of rhodopsin genes. Circles at nodes represent relative bootstrap values, the larger the circles, the higher the bootstrap value (only values >60% are shown); PR: proteorhodopsin; XR: xanthorhodopsin; SR: sensory rhodopsin; highlighted in red is assembled genes in this study; *P. donghaiense* rhodopsin is highlighted in large font size. The heatmap imposed outer of the tree shows the relative expression of each type of rhodopsin in T0, T1, T2, and T3. The outermost dark blue column shows the average FPKM of each type of rhodopsin over the entire T0 to T3 period. **(C)** Relative expression of rhodopsin in the field samples and cultures measured using RT-qPCR normalized by calmodulin and actin. **(B)** Secondary structure analysis of rhodopsin proteins represented in the bloom metatranscriptome dataset. The 7-transmembrane structure resulted from ProteinPredict run. Highlighted in purple are residues that form retinal binding pocket; in green (7th transmembrane domain) is lysine that links rhodopsin to retinal; in red (the 5th transmembrane domain) is keto-carotenoids binding. The 3rd transmembrane domain contains residues that determine the function and absorbing light spectra: the residue in blue functions for spectral tuning, with L (lucine) specifying green absorbing, which accounted for 70% of the rhodopsin cDNA retrieved, and Q (20%) specifying blue light absorbing (upper right pie chart); the two residues in black and the T in purple in the middle forms a motif that determines the function of the rhodopsin, with D-T-E (aspartate-threonine-glutamate) specifying proton pump, which accounted for 61.1% of the rhodopsin cDNA retrieved (upper left pie chart). The function of motif with I-T-E (isoleucine-threonine-glutamate) and motif with T-T-E (threonine-threonine-glutamate) are still unknown, they accounted for 16.7 and 5.4%, respectively (upper left pie chart).

Energy acquisition via electron transport (photosynthetic reaction centers) in photosynthetic organisms is well known but a photosystem-independent pathway through proton translocation (by the action of rhodopsins) has been discovered more recently (Hohmann-Marriott and Blankenship, 2011). The putative rhodopsin-based light energy acquiring mechanism is known only in bacteria (Béja et al., 2001) and several eukaryotic lineages (Lin et al., 2010; Marchetti et al., 2012). Many of the *P. donghaiense* photosynthesis genes were highly expressed and up-regulated at T123, e.g., carbonic anhydrase, a crucial enzyme for inorganic carbon uptake being up-regulated by 101-folds, and Ribulose-1, 5-bisphosphate carboxylase/oxygenase, an essential enzyme for carbon fixation, up-regulated by twofolds (Figure 2Civ, 3B and Supplementary Dataset 4). Meanwhile, in the *P. donghaiense* bloom as many as 375 rhodopsin genes were found (Supplementary Dataset 5), mainly the proton-pump types proteorhodopsin and xanthorhodopsin (Figure 4A) containing proton pumping and absorbing spectrum tuning residues (Figure 4B). Xanthorhodopsins were most highly represented in the T123 metatranscriptomes (Figure 4A). One of the unigenes (CL1Contig24959), identical to the previously reported *P. donghaiense* xanthorhodopsin (Shi et al., 2015), showed a notable ∼48-fold up-regulation at T123 (Figure 4A, *P* < 0.05). RT-qPCR verified the higher expression pattern and also showed much higher expression at T3 than in cultures (Figure 4C). In xanthobacter, retinal and the carotenoid salinixanthin are used as photoantenna to aid in harvesting light energy for xanthorhodopsin (Balashov et al., 2005). We found strong up-regulation of *P. donghaiense* genes involved in the synthesis of retinal and carotenoid at T123 (Supplementary Dataset 6). The heightened xanthorhodopsin expression during the bloom may be associated with bloom development in the turbid and phosphate-deficient ambient environment, as suggested by results from *P. donghaiense* cultures (Shi et al., 2017). Also highly active during the dinoflagellate bloom were ATPases (Figure 2Civ and Supplementary Dataset 4), enzymes responsible for ATP synthesis and hydrolysis.

#### P Acquisition and Nutrient Recycling

Consistent with the diminishing PO_4_^3-^ in the ambient environment at T123 (Figure 1B), *P. donghaiense* markedly up-regulated genes involved in P uptake, including regular and high-affinity PO_4_^3-^ transporters and numerous genes potentially facilitating the utilization of dissolved organic phosphate (Figure 2Ci and Supplementary Dataset 7). Alkaline phosphatase (AP), hydrolyzing phosphoesters to release PO_4_^3-^ for phytoplankton use (Lin X. et al., 2015), was expressed in *P. donghaiense* at T123 but not detectable at T0 (Figure 2Ci and Supplementary Dataset 7). We also detected up-regulation at T123 of apyrase (Figure 2Ci and Supplementary Dataset 7), a calcium-activated enzyme known in plants to catalyze the hydrolysis of ATP to AMP and phosphate (Thomas et al., 1999). Genes known to facilitate utilization of phosphonates (C-P bond organophosphate), such as 2-aminoethylphosphonate (AEP) – pyruvate transaminase (PhnW), PhnI domain-containing protein, and 6-phosphofructokinase 7 (Phn G) were also detected in *P. donghaiense* with up-regulation at T123 (Figure 2Ci and Supplementary Dataset 7), suggesting intracellular phosphorus recycling to cope with the phosphate-deficiency during the bloom (Cui et al., 2016). Besides, we detected PhnI and PhnG at T123 in *P. donghaiense*, suggesting that this species is possibly able to scavenge some types of phosphonates from the environment, as these genes belong to the Phn operon known to regulate utilization of phosphonates in *Escherichia coli* (Fenner et al., 2013). All these reveal a potentially multi-faceted strategy of *P. donghaiense* to cope with phosphate-deficiency for continued growth. Comparatively, diatoms exhibited a lower phosphate uptake capacity, with only one type of inorganic phosphate transporter highly expressed at T0 (Figure 2Ci and Supplementary Dataset 7).

Our metatranscriptomic data suggest that recycling of intracellular components via autophagy might be an important source of nutrients to support *P. donghaiense* growth. Autophagy is a lysosome-dependent cellular catabolic mechanism, which composes vesicle-associated membrane protein, syntaxin and Glogi SNAP receptor complex member 2 (Renna et al., 2011). Activation of autophagy requires autophagy-related protein 8 (ATG8) in algae (Pérez-Pérez et al., 2010); ATG12 and ATG3 also play an essential role in autophagosome formation (Pérez-Pérez et al., 2010). These genes were more highly expressed in *P. donghaiense* at T123 (Figure 2Ci and Supplementary Dataset 7). In comparison, although diatom autophagy-related genes were up-regulated at T0, the number of genes and the magnitude of expression increase were lower than dinoflagellates at T123 (Figure 2Ci). More diversified phosphate acquisition strategy and the recycling of intracellular components via autophagy found in our study potentially in part define the ecological niche to acquire nutrients for *P. donghaiense* to form the bloom.

#### Antimicrobial Defense

An effective defense mechanism may be important for population growth and bloom formation (Potin et al., 2002; Gobler et al., 2011). We found nine *P. donghaiense* genes in the bloom metatranscriptome annotated as antimicrobial peptides (Supplementary Dataset 8). Among these, macrophage migration inhibitory factor (MIF) is known in humans and animals as a pivotal regulator of innate immunity and an integral component of the host antimicrobial alarm system and stress response (Kleemann et al., 2000). Plant defensins are generally recognized as host defense peptides against some pathogenic fungi and bacteria (Broekaert et al., 1995). Synaptobrevin is one of the SNARE proteins involved in the formation of SNARE complexes while pleurodin and SNARE are peptides that have antibacterial activity in invertebrates and vertebrates (Collins et al., 2003; Smith et al., 2010). The expression of all these gene homologs was undetectable at T0 but substantial at T123 as well as in the other bloom mentioned above (Supplementary Dataset 8), indicative of potential importance of antimicrobial defense in natural bloom formation. It is noteworthy that a functionally similar (but with distinct gene sets) antimicrobial defense mechanism has been identified in the genome of *Aureococcus anophagefferens* prone to form massive blooms (Gobler et al., 2011). Whether these mechanisms equip the bloom algae to fight off algicidal bacteria will be of high interest to investigate in the future.

#### Metabolism Regulated by Novel and Unique Genes

Six novel genes were identified as some of the most highly expressed genes in *P. donghaiense* (Supplementary Dataset 9). Four of these showed 10- to over 100-fold elevation of expression during T123 (Unknown I), and were also expressed actively in the other *P. donghaiense* bloom mentioned above (Supplementary Datasets 9, 10). Two of them showed an opposite expression pattern, dramatically up-regulated at T0 (Unknown II, Supplementary Dataset 9). These genes are likely unique genes important in regulating dinoflagellate population proliferation and bloom formation (Unknown I) or population maintenance (Unknown II). It is noteworthy that major basic nuclear proteins (MBNPs) were also highly expressed at T123 (Figure 3B and Supplementary Dataset 9). The function of MBNPs in dinoflagellates is unclear but is thought to replace histones in packaging chromosomal DNA (Kato et al., 1997). The high expression of MBNPs at T123 suggests some important role in cell proliferation. Many of the highly expressed genes reported here have never been detected, or never found to be highly expressed, in culture studies, underscoring the need for more *in situ* studies in order to fully understand underlying mechanisms of competition and succession of phytoplankton lineages in the natural assemblages.

### Integrative and Future Perspectives on Understanding Diatom-to-Dinoflagellate Regime Shift and Potential Drivers of *P. donghaiense* Bloom

**FIGURE 5:**
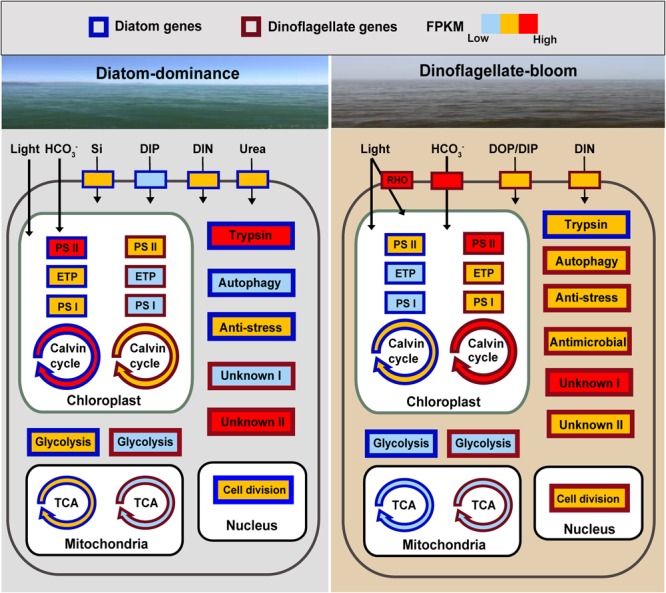
Schematic summary of changes in expression of key genes or metabolic processes in diatoms and the dinoflagellate *P. donghaiense* in the course of the diatom-dominance to dinoflagellate-bloom regime shift. RHO, rhodopsin; ETP, electron transfer proteins; Genes or pathways with FPKM ranging from 0 to 100 are shown in light blue (low activity), from 100 to 1000 in orange, over 1000 in red (high activity). Generally, FPKM of pathways involving multiple genes are based on the gene with the highest FPKM. DIP (dissolved inorganic phosphate) is indicated in light blue in diatoms during diatom-dominance because it only composed one type of transporter.

Our results provide insights into molecular inner working of a phytoplankton community during a shift from diatom (*Skeletonema*)-dominance to a dinoflagellate (*P. donghaiense*)-bloom. Diatoms and dinoflagellates may use the same biological processes when at high relative abundance in their common coastal environment. This is evident from the similar set of most active metabolic processes (energy and nutrient acquisition, anti-stress) to promote growth during dominance (Figure 5). More importantly, our data also reveal distinct metabolic pathways between diatoms (carbohydrate metabolism, trypsin-based metabolism) and dinoflagellates (proton-pump rhodopsin-based energy acquisition, antimicrobial defense) and many highly expressed unique complements of functional genes, potentially defining distinct ecological niches for these two lineages. *P. donghaiense* possessed more diversified light energy and phosphate acquisition strategy and antimicrobial defense, which might lead them to outgrow diatoms and form blooms (Figure 5). “Omic” studies often lead to discoveries of unsuspected physiologies or biochemistries underpinning certain ecological phenotype, but typically the discoveries remain to be proven in subsequent experimental studies. With no exception, many of the findings reported here stand as new questions and hypotheses for further experimental inquiries, with functions of key genes to be demonstrated using a functional genetic tool. While no functional genetic tool is yet available, efforts to develop one are underway in various laboratories. Finally, many of the highly expressed genes reported here have never been detected, or never found to be highly expressed in culture studies, underscoring the need for more *in situ* studies in order to fully understand underpinning mechanisms of competition and succession of phytoplankton lineages in the natural assemblages.

## Author Contributions

SL and YZ conceived the study. SL, YZ, XL, and XS drafted and edited the manuscript and figures. YZ, LXL, and XS performed the bioinformatics analyses. YZ, HL, and LL performed the experiments. All authors read and approved the final version of the manuscript.

## Conflict of Interest Statement

The authors declare that the research was conducted in the absence of any commercial or financial relationships that could be construed as a potential conflict of interest.

## Footnotes

1 ftp://ftp.ncbi.nlm.nih.gov/blast/db/ [accessed 03, 2015]
