# Supplementary material for: Metatranscriptomic Signatures Associated With Phytoplankton Regime Shift From Diatom Dominance to a Dinoflagellate Bloom
Source: Front Microbiol. 2019 Mar 22;10:590. doi: 10.3389/fmicb.2019.00590 (PMC6439486; doi:10.3389/fmicb.2019.00590)
Supplement: Supplementary file 1 [file Table_1.docx]

**Manuscript title:**

Metatranscriptomic signatures associated with regime shift

from diatom dominance to dinoflagellate bloom

**Authorship:**

Yaqun Zhang^a^, Xin Lin^a^, Xinguo Shi^a,b^, Lingxiao Lin^a^, Hao Luo^a^, Ling Li^a^, Senjie Lin ^a, c*^

1. *State Key Laboratory of Marine Environmental Science and College of Ocean and Earth Sciences, Xiamen University, Xiamen 361102, China*
2. *College of Biological Science and Engineering, Fuzhou University, Fuzhou, 350108, China*
3. *Department of Marine Sciences, University of Connecticut, Groton, CT 06340, USA*

**SI including:**

Figure S1-S6; Table S1-S2; Dataset 1-10

**Supplementary Figures and legends (Figure S1-S66)**

**
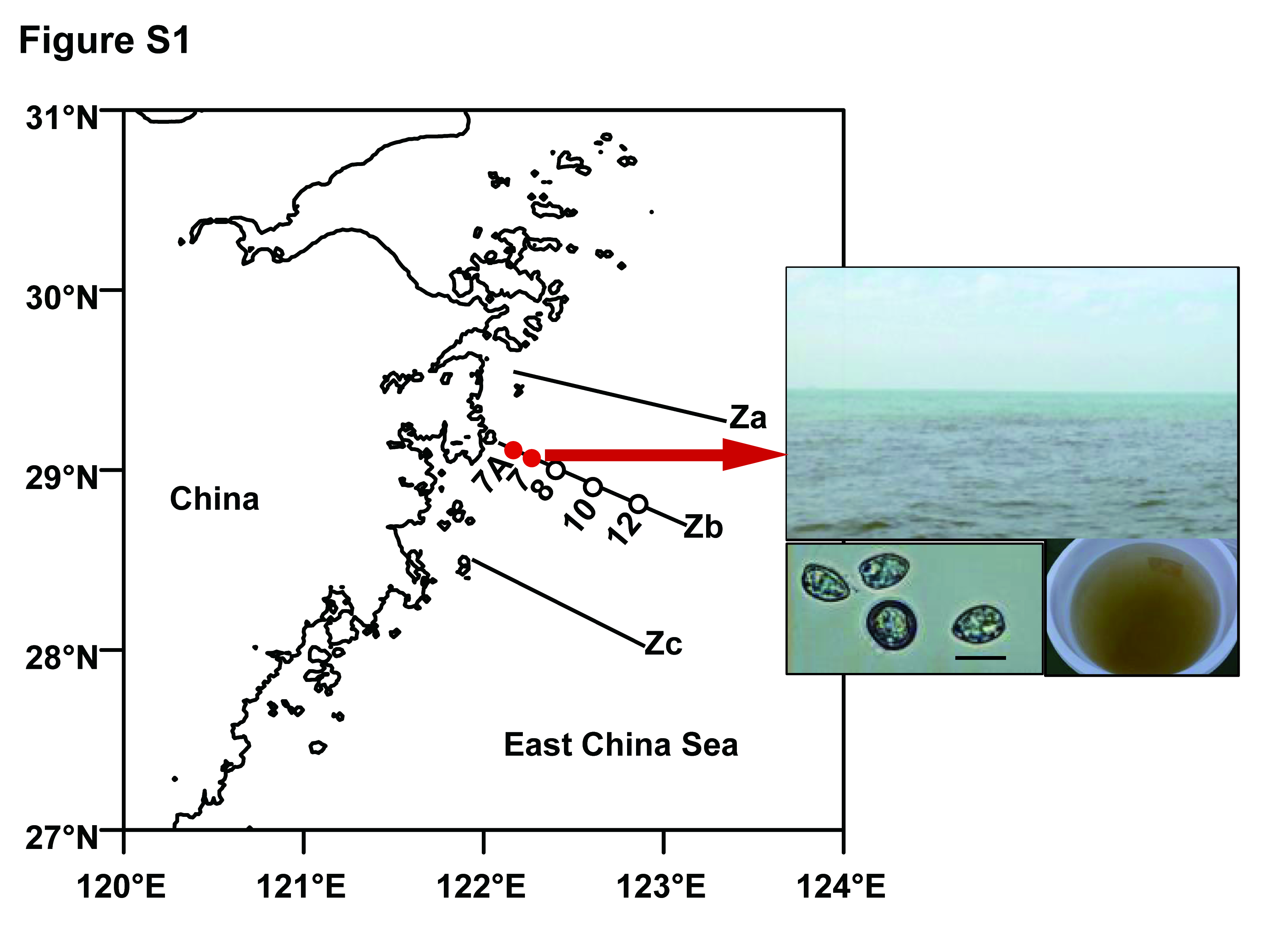
**

**Figure S1.** Sampling sites of the 2014 cruise, photographs of the red-tide bloom and microscopic image of the causative species *P. donghaiense* (Inset; scale=20 μm).


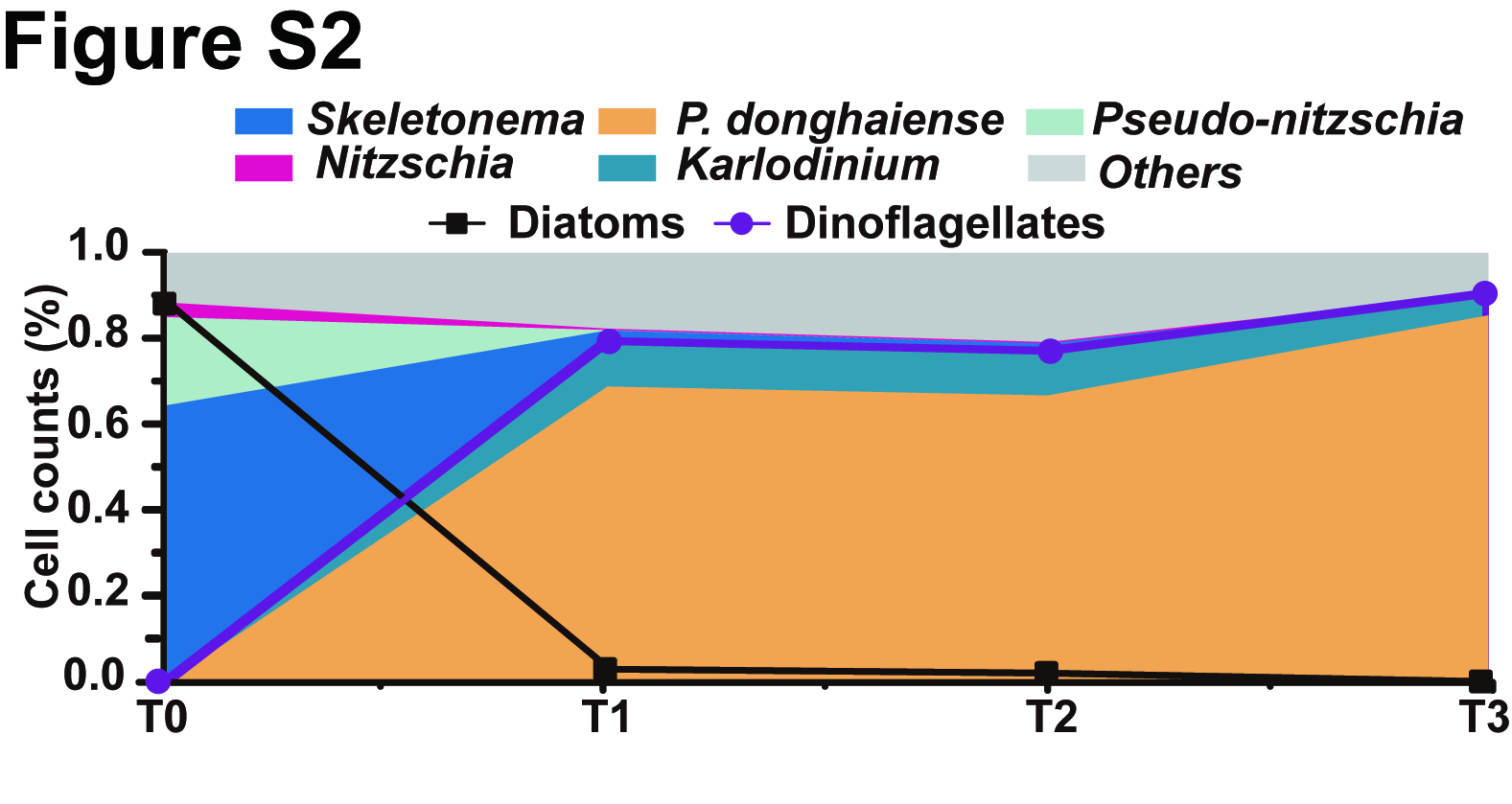


**Figure S2**. Cell concentrations of major diatom and dinoflagellate species and proportions of diatoms (black line) and dinoflagellates (purple line) in the total community.


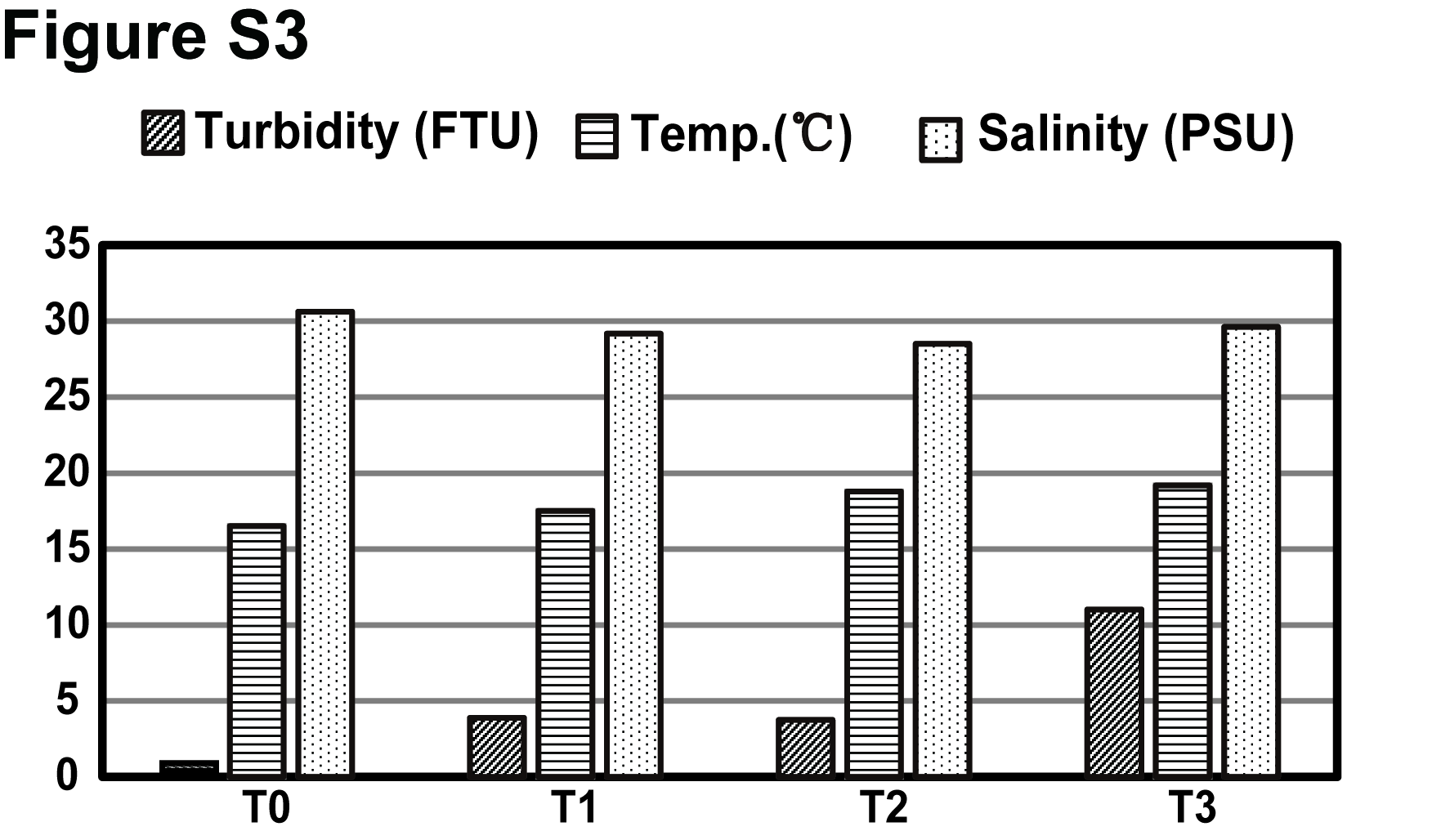


**Figure S3.** Environmental parameters measured at the sampling sites.


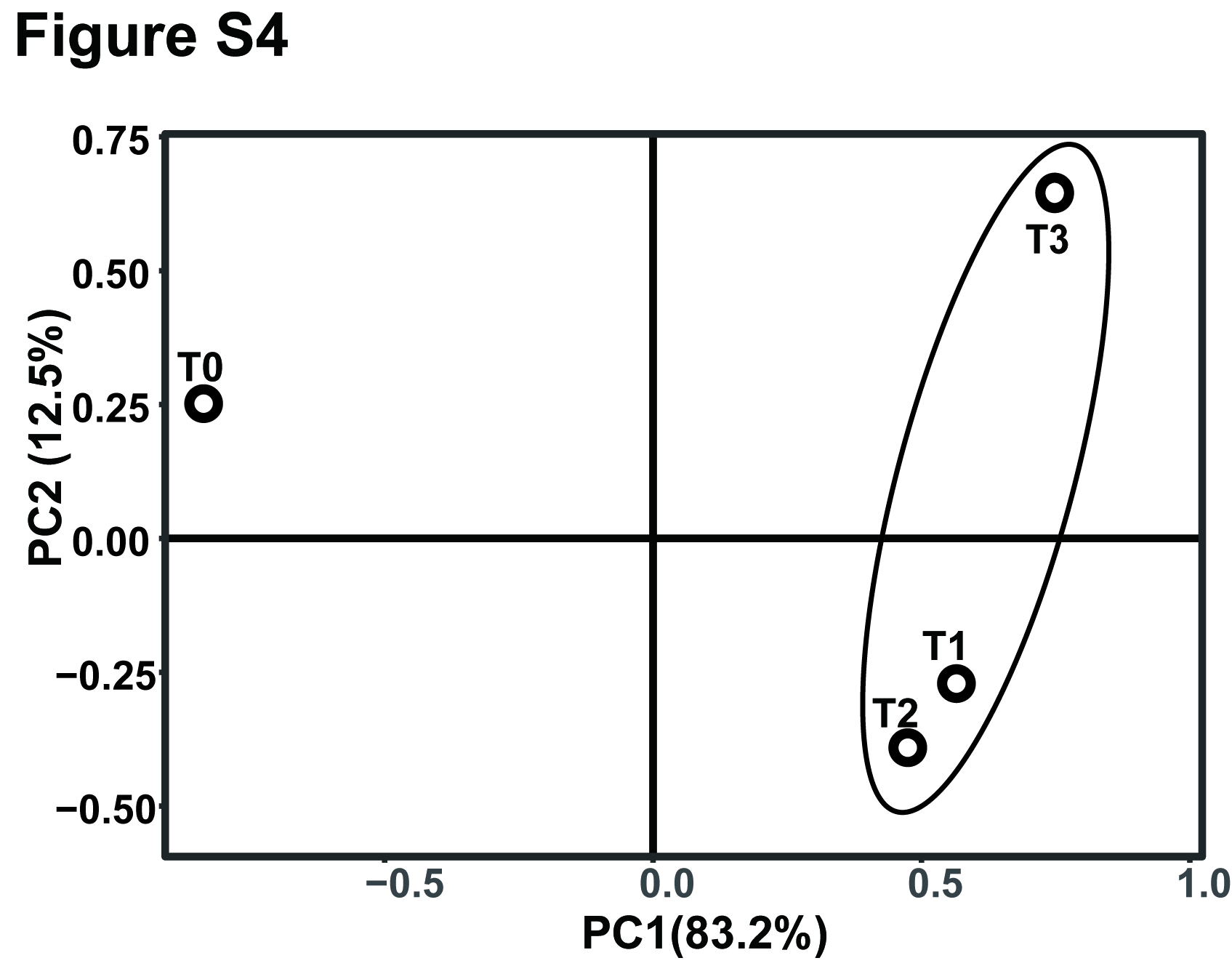


**Figure S4.** PCA of the FPKM values across the four time-point samples.


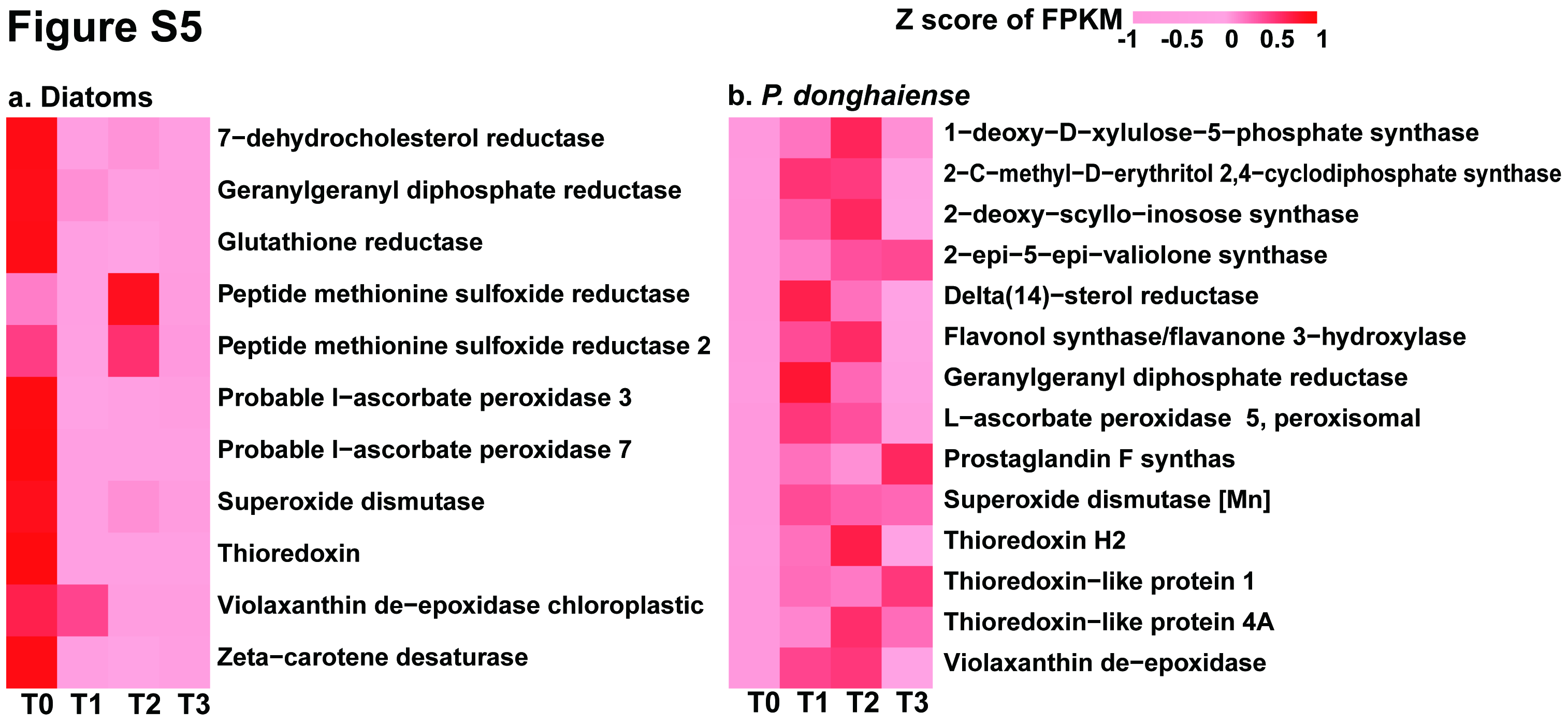


**Figure S5**. Expression levels of anti-stress genes in diatoms (a) and *P. donghaiense* (b).


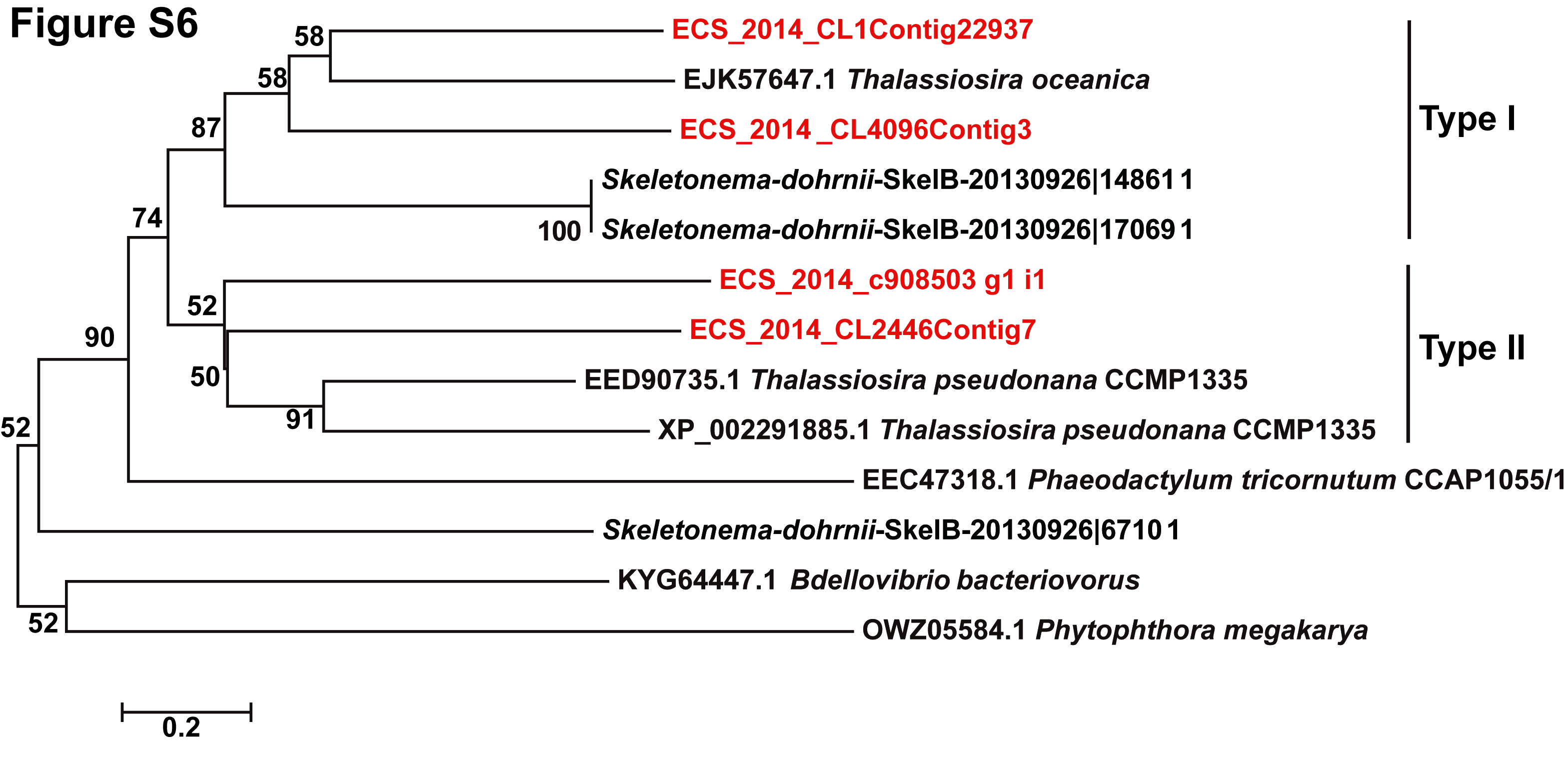


**Figure S6**. Phylogenetic analysis of trypsins showing their affiliation with previously documented diatom homologs. Highlighted in red are representative assembled transcripts in this study.

**Supplemental Table S1-S2**

| Table S1. Sequences of primes used in this study. | | |
| --- | --- | --- |
| ***Primer*** | ***Sequence*** |  |
| Rhodopsin-QF | ACTACGGCGAACTGACTGTG |  |
| Rhodopsin-QR | TCGAACACAATGCACAAGAA |  |
| Actin-QF | GGACCAGAAGGACAGCTACG |  |
| Actin-QR | CATGTCGTCCCAGTTGGTCA |  |
| Cal-QF | CGGAGTTCAAGGAGGCGTTCTC |  |
| Cal-QR | CGTCCACCTCGTTGATCATGTCTT |  |

Table S2. Statistics of metatranscriptome Hiseq 2000 sequencing of the four field samples.

|  | **T0** | **T1** | **T2** | **T3** |
| --- | --- | --- | --- | --- |
| Approximate total size (bp) | 10,203,335,000 | 12,990,992,250 | 12,921,527,250 | 14,262,063,250 |
| Raw sequence reads | 81,626,680 | 103,927,938 | 103,372,218 | 114,096,506 |
| Trimmed sequence reads | 69,101,500 | 88,074,572 | 86,474,676 | 95,959,994 |
| Average read length (bp) | 123.85 | 123.94 | 123.96 | 123.9 |
| Assembled contigs | 916,311 | 720,875 | 665,788 | 379,482 |
| Contigs average length (bp) | 352.14 | 335.02 | 337.81 | 337.81 |
| (C+G)% content | 49.64 | 54.95 | 52.6 | 53.18 |
| Total predicted unigenes | 416,844 | 263,750 | 249,364 | 179,669 |
| Unigenes with functional annotation |  |  |  |  |
| Nr | 185980 | 93240 | 87854 | 61255 |
| COG | 65175 | 38326 | 35898 | 25132 |
| GO | 81376 | 39769 | 36791 | 23866 |
| KO | 70856 | 41469 | 38982 | 26511 |
| KEGG pathway | 52991 | 31242 | 29310 | 19624 |
| Pfam | 21018 | 21062 | 20545 | 16565 |
